# Supplementary material for: What are the Andean Colombian anurans? Empirical regionalization proposals vs. observed patterns of compositional dissimilarity
Source: PeerJ. 2023 Jun 13;11:e15217. doi: 10.7717/peerj.15217 (PMC10274619; doi:10.7717/peerj.15217)
Supplement: Supplemental Information 1 [file peerj-11-15217-s001.doc]

**Table S1:** Distribution information sources per family. For a complete list of records per species please contact with the authors.

| **Family (species number)** | **Sources** |
| --- | --- |
| Aromobatidae (8) | Anganoy-Criollo, 2012 |
|  | Anganoy-Criollo, 2013 |
|  | Barrio-Amorós and Santos, 2012 |
|  | Cochran and Goin, 1970 |
|  | Coleccíon de Herpetología de la Universidad del Valle, 2016 |
|  | Frost, 2018 |
|  | Grant and Rodríguez, 2001 |
|  | Grant et al., 2006 |
|  | Grant et al., 2007 |
|  | IAvH, 2018 |
|  | Restrepo et al., 2017 |
|  | Rivero and Serna, 1995 |
|  | Sanchez, 2013 |
|  | Sánchez, 2013 |
|  | Serna-Botero and Ramírez-Castaño, 2017 |
|  | Silverstone, 1976 |
| Bufonidae (63) | Acosta Galvis et al., 2006 |
|  | Acosta-Galvis et al., 2006 |
|  | Ardila-Robayo and Ruiz-Carranza, 1998 |
|  | Ardila-Robayo, 1999 |
|  | Bernal et al., 2005 |
|  | Bravo-Valencia and Rivera-Correa, 2011 |
|  | Canatella, 1981 |
|  | Castro-Herrera and Vargas-Salinas, 2008 |
|  | Cisneros-Heredia and Gluesekamp, 2010 |
|  | Cochran and Goin, 1970 |
|  | Coleccíon de Herpetología de la Universidad del Valle, 2016 |
|  | Coloma et al., 2010 |
|  | Encizo-Calle et al. 2017 |
|  | Frost, 2017 |
|  | Grant and Bolívar, 2014 |
|  | Grant, 1999 |
|  | Hernández-Córdoba et al, 2014 |
|  | IAvH, 2018 |
|  | ICN, 2004 |
|  | Kattan, 1986 |
|  | La Marca et al. 1989 |
|  | Lynch and Renjifo, 1990 |
|  | Lynch and Ruiz-Carranza, 1981 |
|  | Lynch, 1993a |
|  | Lynch, 2006 |
|  | Mueses Cisneros et al., 2012 |
|  | Mueses Cisneros, 2003 |
|  | Mueses Cisneros, 2009 |
|  | Narvaes and Trefaut-Rodrigues, 2009 |
|  | Osorno Muñoz et al., 2001 |
|  | Pramuk, 2006 |
|  | Restrepo et al., 2017 |
|  | Riuz Carranza and Osorno Muñoz, 1994 |
|  | Rivero and Castaño, 1990 |
|  | Rivero and Serna, 1991 |
|  | Rivero, 1963 |
|  | Ron et al., 2015 |
|  | Ruiz-Carranza and Hernández-Camacho, 1976a |
|  | Ruiz-Carranza and Hernández-Camacho, 1978 |
|  | Ruiz-Carranza et al., 1995 |
|  | Serna-Botero and Ramírez-Castaño, 2017 |
|  | Trueb, 1971 |
|  | Vanegas Guerrero et al., 2016 |
|  | Vélez Rodríguez and Ruiz-Carranza, 1997 |
|  | Vélez Rodríguez and Ruiz-Carranza, 2002 |
|  | Vélez Rodríguez, 1999 |
| Centrolenidae (69) | Acosta-Galvis, 2012 |
|  | Acosta-Galvis, 2017 |
|  | Andrade and Lynch, 2007 |
|  | Bernal et al., 2005 |
|  | Bolívar et al, 1999 |
|  | Cisneros-Heredia and McDiarmid, 2007 |
|  | Cochran and Goin, 1970 |
|  | Coleccíon de Herpetología de la Universidad del Valle, 2016 |
|  | Daza and Barrientos, 2005 |
|  | Duellman and Burrowes, 1989 |
|  | Duellman, 1980 |
|  | Duellman, 1981 |
|  | Frost, 2017 |
|  | Gutiérrez-Cárdenas, 2005 |
|  | IAvH, 2018 |
|  | ICN, 2004 |
|  | Jaramillo et al., 2015 |
|  | Lynch and Duellman, 1973 |
|  | Lynch and Ruiz-Carranza, 1996 |
|  | Lynch, 1990a |
|  | Lynch, 1993 |
|  | Lynch, 2001 |
|  | Malambo et al., 2013 |
|  | Malambo et al., 2017 |
|  | Molina Zuluaga et al., 2017 |
|  | Mueses Cisneros and Moreno-Quintero, 2012 |
|  | Mueses Cisneros, 2005a |
|  | Noble, 1920 |
|  | Osorio Dominguez and Quintero Angel, 2012 |
|  | Rada and Guayasamin, 2008 |
|  | Rada et al., 2007 |
|  | Rada et al., 2017 |
|  | Restrepo et al., 2017 |
|  | Rivera Correa, 2010 |
|  | Rivero, 1985 |
|  | Rojas et al., 2014 |
|  | Ruiz and Lynch, 1998 |
|  | Ruiz-Carranza and Lynch, 1991 |
|  | Ruiz-Carranza and Lynch, 1991a |
|  | Ruiz-Carranza and Lynch, 1991b |
|  | Ruiz-Carranza and Lynch, 1995 |
|  | Ruiz-Carranza and Lynch, 1995a |
|  | Ruiz-Carranza and Lynch, 1995b |
|  | Ruiz-Carranza and Lynch, 1995c |
|  | Ruiz-Carranza and Lynch, 1996 |
|  | Ruiz-Carranza and Lynch, 1997 |
|  | Ruiz-Carranza and Lynch, 1998 |
|  | Ruiz-Carranza et al., 1986 |
|  | Serna-Botero and Ramírez-Castaño, 2017 |
|  | Suárez Badillo and Ramírez, 2004 |
|  | Suárez Mayorga, 1999 |
|  | Velásquez Alvárez et al., 2007 |
|  | Wild, 1994 |
| Ceratophryidae (1) | Cochran and Goin, 1970 |
|  | IAvH, 2018 |
| Craugastoridae (224) | Acevedo et al., 2014 |
|  | Acosta-Galvis et al., 2006 |
|  | Acosta-Galvis, 2015 |
|  | Acosta-Galvis, 2017 |
|  | Andrade and Lynch, 2007 |
|  | Anganoy-Criollo and Ramírez, 2017 |
|  | Bernal et al., 2005 |
|  | Bolívar et al., 2011 |
|  | Boulenger, 1896 |
|  | Boulenger, 1908 |
|  | Boulenger, 1912 |
|  | Buitrago-González et al., 2016 |
|  | Cochran and Goin, 1970 |
|  | Coleccíon de Herpetología de la Universidad del Valle, 2016 |
|  | Duarte-Marín et al., 2018 |
|  | Duellman and Simmons, 1977 |
|  | Fernández-Roldán, 2014 |
|  | Frost, 2017 |
|  | Frost, 2018 |
|  | Galeano and Urbina, 2004 |
|  | García and Lynch, 2006 |
|  | Goin and Cochran, 1963 |
|  | González-Durán et al, 2017 |
|  | González-Durán et al., 2017 |
|  | González-Durán, 2016 |
|  | Guayasamin et al., 2015 |
|  | IAvH, 2018 |
|  | ICN, 2004 |
|  | Jeréz et al., 2001 |
|  | Lynch and Ardila-Robayo, 1999 |
|  | Lynch and Ardila-Robayo, 2004 |
|  | Lynch and Burrowes, 1990 |
|  | Lynch and Duellman, 1980 |
|  | Lynch and Myers, 1983 |
|  | Lynch and Rueda Almonacid, 1997 |
|  | Lynch and Rueda Almonacid, 1998 |
|  | Lynch and Rueda Almonacid, 1999 |
|  | Lynch and Ruiz-Carranza, 1982 |
|  | Lynch and Ruiz-Carranza, 1983 |
|  | Lynch and Ruiz-Carranza, 1996 |
|  | Lynch and Suárez-Mayorga, 2000 |
|  | Lynch and Suárez-Mayorga, 2003 |
|  | Lynch et al., 1994 |
|  | Lynch et al., 1996 |
|  | Lynch, 1895 |
|  | Lynch, 1973 |
|  | Lynch, 1975 |
|  | Lynch, 1975a |
|  | Lynch, 1976 |
|  | Lynch, 1976a |
|  | Lynch, 1977 |
|  | Lynch, 1977a |
|  | Lynch, 1978 |
|  | Lynch, 1980 |
|  | Lynch, 1980a |
|  | Lynch, 1980b |
|  | Lynch, 1981 |
|  | Lynch, 1981a |
|  | Lynch, 1981b |
|  | Lynch, 1983 |
|  | Lynch, 1984 |
|  | Lynch, 1984a |
|  | Lynch, 1986 |
|  | Lynch, 1989a |
|  | Lynch, 1990 |
|  | Lynch, 1991 |
|  | Lynch, 1992 |
|  | Lynch, 1992a |
|  | Lynch, 1992b |
|  | Lynch, 1992c |
|  | Lynch, 1994 |
|  | Lynch, 1994a |
|  | Lynch, 1994b |
|  | Lynch, 1995 |
|  | Lynch, 1996 |
|  | Lynch, 1996a |
|  | Lynch, 1997 |
|  | Lynch, 1998 |
|  | Lynch, 1998a |
|  | Lynch, 1999 |
|  | Lynch, 2000 |
|  | Lynch, 2001 |
|  | Lynch, 2003 |
|  | Lynch, 2003a |
|  | Lynch, 2003b |
|  | Lynch, 2006 |
|  | Malambo and Madrid, 2008 |
|  | Meneses-Pelayo et al., 2017 |
|  | Meza-Joya, 2016 |
|  | Mueses Cisneros and Moreno-Quintero, 2012 |
|  | Mueses Cisneros et al., 2013 |
|  | Mueses Cisneros, 2005a |
|  | Mueses Cisneros, 2007a |
|  | Muñoz-Arcos et al., 2016 |
|  | Ortega-Andrade y Vanegas, 2014 |
|  | Ospina Sarria et al., 2015 |
|  | Ospina Sarria, et al., 2015 |
|  | Ospina-Sarria et al, 2011 |
|  | Ospina-Sarria et al., 2011 |
|  | Pyburn and Lynch, 1981 |
|  | Restrepo et al., 2017 |
|  | Restrepo, et al., 2017 |
|  | Rivera et al., 2017 |
|  | Rivera-Correa and Daza, 2016 |
|  | Rivera-Correa et al., 2016 |
|  | Rivera-Correa et al., 2017 |
|  | Rivera-Prieto, Rivera-Correa and Daza, 2014 |
|  | Rivero and Serna, 1988 |
|  | Roa Trujillo and Ruiz-Carranza, 1991 |
|  | Rojas Rivera et al., 2013 |
|  | Romero García et al., 2015 |
|  | Rueda Almónacid and Lynch, 1983 |
|  | Rueda Almonacid et al., 2003 |
|  | Ruiz-Carranza and Lynch, 1997 |
|  | Ruiz-Carranza et al., 1997 |
|  | Serna-Botero and Ramírez-Castaño, 2017 |
|  | Suárez Badillo and Ramírez, 2004 |
|  | Valencia-Zuleta et al., 2016 |
|  | Velandia Perilla et al., 2011 |
| Dendrobatidae (67) | Acosta-Galvis and Pinzón, 2018 |
|  | Acosta-Galvis and Vargas-Ramírez, 2018 |
|  | Acosta-Galvis et al., 2006 |
|  | Acosta-Galvis, 2012 |
|  | Acosta-Galvis, 2017 |
|  | Amézquita et al., 2013 |
|  | Andrade and Lynch, 2007 |
|  | Anganoy-Criollo, 2013 |
|  | Ardila-Robayo et al., 1999 |
|  | Bernal et al., 2005 |
|  | Boulenger, 1899 |
|  | Cochran and Goin, 1970 |
|  | Coleccíon de Herpetología de la Universidad del Valle, 2016 |
|  | Frost, 2017 |
|  | Frost, 2018 |
|  | Grant and Ardila-Robayo, 2002 |
|  | Grant and Castro-Herrera, 1998 |
|  | Grant and Castro, 1998 |
|  | Grant and Myers, 2013 |
|  | Grant et al., 1997 |
|  | Grant et al., 2006 |
|  | Grant et al., 2017 |
|  | Grant, 2004 |
|  | Grant, 2007 |
|  | IAvH, 2018 |
|  | ICN, 2004 |
|  | Lynch, 1982 |
|  | Marín-Castaño et al., 2018 |
|  | Márquez et al., 2017 |
|  | Mueses Cisneros, Cepeda-Quilindo and Moreno-Quintero, 2008 |
|  | Myers and Burrowes, 1987 |
|  | Myers and Daly, 1976 |
|  | Myers and Daly, 1980 |
|  | Myers, 1991 |
|  | Ramos et al., 2018 |
|  | Restrepo et al., 2017 |
|  | Rivero and Granados-Díaz, 1990 |
|  | Rivero and Serna, 1986 |
|  | Rivero and Serna, 1991a |
|  | Rivero and Serna, 1995 |
|  | Rueda Almonacid et al., 2006 |
|  | Ruiz-Carranza and Ramírez-Pinilla, 1992 |
|  | Sanchez, 2013 |
|  | Sánchez, 2013 |
|  | Serna-Botero and Ramírez-Castaño, 2017 |
|  | Silverstone, 1971 |
|  | Silverstone, 1975 |
|  | Silverstone, 1975a |
|  | Silverstone, 1976 |
| Eleutherodactylidae (3) | Acosta-Galvis et al., 2006 |
|  | Coleccíon de Herpetología de la Universidad del Valle, 2016 |
|  | IAvH, 2018 |
|  | ICN, 2004 |
|  | Jiménez-Rivillas et al., 2013 |
|  | Lynch, 2001a |
|  | Restrepo et al., 2017 |
|  | Serna-Botero and Ramírez-Castaño, 2017 |
| Hemiphractidae (25) | Bernal et al., 2005 |
|  | Cochran and Goin, 1970 |
|  | Coleccíon de Herpetología de la Universidad del Valle, 2016 |
|  | Duellman and Burrowes, 1986 |
|  | Duellman and Hillis, 1987 |
|  | Duellman and Pyles, 1980 |
|  | Duellman, 1983 |
|  | Duellman, 1987 |
|  | Gaige, 1933 |
|  | Hill et al., 2018 |
|  | IAvH, 2018 |
|  | ICN, 2004 |
|  | Lynch, 2008 |
|  | Mueses Cisneros, 2005 |
|  | Mueses Cisneros, 2005a |
|  | Ruiz-Carranza and Hernández-Camacho, 1976 |
|  | Ruiz-Carranza et al., 1997 |
|  | Serna-Botero and Ramírez-Castaño, 2017 |
|  | Sheil and Mendelson, 2001 |
|  | Sheil et al., 2001 |
|  | Trueb, 1974 |
| Hylidae (87) | Acosta-Galvis et al., 2006 |
|  | Acosta-Galvis, 2012 |
|  | Acosta-Galvis, 2017 |
|  | Acosta-Galvis, 2018 |
|  | Ardila-Robayo and Ruiz-Carranza, 1993 |
|  | Arenas-Rodríguez et al., 2018 |
|  | Barrio-Amorós et al., 2004 |
|  | Barrio-Amorós et al., 2006 |
|  | Bernal et al., 2005 |
|  | Cochran and Goin, 1970 |
|  | Coleccíon de Herpetología de la Universidad del Valle, 2016 |
|  | Duellman and Altig, 1978 |
|  | Duellman and Berger, 1982 |
|  | Duellman and Crump, 1974 |
|  | Duellman and Fouquette, 1968 |
|  | Duellman and Hillis, 1990 |
|  | Duellman and Trueb, 1983 |
|  | Duellman, 1971 |
|  | Duellman, 1972 |
|  | Duellman, 1972a |
|  | Duellman, 1973 |
|  | Duellman, 1989 |
|  | Echavarría-Rentería et al., 2015 |
|  | Faivovich et al., 2006 |
|  | Frost, 2018 |
|  | Guarnizo, Escallón, Cannatella, and Amézquita, 2012 |
|  | Gutiérrez-Cárdenas et al., 2013 |
|  | IAvH, 2018 |
|  | ICN, 2004 |
|  | Jungfer et al., 2013 |
|  | Kaplan and Ruiz-Carranza, 1997 |
|  | Kaplan, 1991 |
|  | Kaplan, 1994 |
|  | Kaplan, 1997 |
|  | Kluge, 1979 |
|  | Lynch and Suárez-Mayorga, 2001 |
|  | Lynch and Suárez-Mayorga, 2011 |
|  | Lynch, 2006 |
|  | Malambo-L and Madrid-Ordóñez, 2008 |
|  | Méndez-Narváez et al., 2014 |
|  | Montezuma and Mueses Cisneros, 2009 |
|  | Mueses Cisneros and Anganoy-Criollo, 2008 |
|  | Mueses Cisneros and Moreno-Quintero, 2012 |
|  | Mueses Cisneros and Perdomo-Castillo, 2011 |
|  | Mueses Cisneros, 2005a |
|  | Myers and Duellman, 1982 |
|  | Pérez-Villota et al., 2009 |
|  | Pyburn and Fouquette, 1971 |
|  | Ramírez-Chaves et al., 2018 |
|  | Restrepo et al., 2017 |
|  | Restrepo, et al., 2017 |
|  | Rivera-Correa and Faivovich, 2013 |
|  | Rivera-Correa and Gutiérrez-Cárdenas, 2012 |
|  | Rivera-Correa and Orrico, 2013 |
|  | Rojas-Runjaic et al., 2018 |
|  | Ruiz-Carranza and Ardila-Robayo, 1991 |
|  | Ruiz-Carranza and Lynch, 1982 |
|  | Sánchez, 2010 |
|  | Serna-Botero and Ramírez-Castaño, 2017 |
|  | Smith and Noonan, 2001 |
|  | Trueb and Duellman, 1971 |
| Leptodactylidae (26) | Acosta-Galvis et al., 2006 |
|  | Acosta-Galvis, 2012 |
|  | Acosta-Galvis, 2017 |
|  | Bernal et al., 2005 |
|  | Canatella and Duellman, 1984 |
|  | Cochran and Goin, 1970 |
|  | Coleccíon de Herpetología de la Universidad del Valle, 2016 |
|  | Heyer and De Sá, 2011 |
|  | Heyer, 1973 |
|  | Heyer, 1978 |
|  | Heyer, 1979 |
|  | Heyer, 1994 |
|  | Heyer, 1997 |
|  | Heyer, 2005 |
|  | IAvH, 2018 |
|  | ICN, 2004 |
|  | Lynch, 1989 |
|  | Lynch, 2001a |
|  | Lynch, 2006 |
|  | Méndez-Narváez, et al., 2009 |
|  | Mueses Cisneros and Moreno-Quintero, 2012 |
|  | Restrepo et al., 2017 |
|  | Roberto et al., 2013 |
|  | Serna-Botero and Ramírez-Castaño, 2017 |
| Microhylidae (6) | Acosta-Galvis et al., 2006 |
|  | Acosta-Galvis, 2012 |
|  | Bernal et al., 2005 |
|  | Cochran and Goin, 1970 |
|  | Coleccíon de Herpetología de la Universidad del Valle, 2016 |
|  | IAvH, 2018 |
|  | ICN, 2004 |
|  | Mueses Cisneros and Moreno-Quintero, 2012 |
| Phyllomedusidae (11) | Acosta-Galvis et al., 2006 |
|  | Acosta-Galvis, 2017 |
|  | Andrade and Lynch, 2007 |
|  | Barrio-Amorós, 2006 |
|  | Canatella, 1980 |
|  | Cochran and Goin, 1970 |
|  | Coleccíon de Herpetología de la Universidad del Valle, 2016 |
|  | Duellman, 1974 |
|  | IAvH, 2018 |
|  | ICN, 2004 |
|  | Lynch and Suárez-Mayorga, 2011 |
|  | Lynch, 2006 |
|  | Ortega-Andrade, 2008 |
|  | Restrepo et al., 2017 |
|  | Rivera-Correa et al., 2013 |
|  | Serna-Botero and Ramírez-Castaño, 2017 |
|  | Vargas and Gutiérrez-Cárdenas, 2005 |
| Pipidae (2) | Cochran and Goin, 1970 |
|  | IAvH, 2018 |
|  | ICN, 2004 |
|  | Trueb, 1984 |
| Ranidae (2) | Acosta Galvis, 1999 |
|  | Cochran and Goin, 1970 |
|  | Coleccíon de Herpetología de la Universidad del Valle, 2016 |
|  | Hillis and de Sá, 1988 |
|  | IAvH, 2018 |
|  | Serna-Botero and Ramírez-Castaño, 2017 |

**References**

Acevedo A, Pallares RF, Perez KS. 2014. Nuevos registros de especies del género *Pristimantis* (Anura: Craugastoridae) para el nororiente de Colombia. *Revista Biodiversidad Neotropical* 4:162-169.

Acosta-Galvis AR. 2018. Una nueva rana de huesos verdes del género *Scinax* (Anura: Hylidae) asociada a los bosques subandinos de la cuenca del río Magdalena, Colombia. *Biota Colombiana* 19(Sup 1):131-159.

Acosta-Galvis AR. 1999. Distribución variación y estatus taxonómico de las poblaciones del complejo *Rana palmipes* (Amphibia: Anura: Ranidae) en Colombia. *Revista de la Academia Colombiana de Ciencias Exactas, Físicas y Naturales* 23:215-224.

Acosta-Galvis AR. 2012. Anfibios de los enclaves secos en la ecorregión de La Tatacoa y su área de influencia, alto Magdalena, Colombia. *Biota Colombiana*, 13:182-210.

Acosta-Galvis AR. 2015. Una nueva especie del género *Pristimantis* (Anura: Craugastoridae) del complejo de páramos Merchán-Iguaque (Boyacá, Colombia). *Biota Colombiana* 16:107-127.

Acosta-Galvis AR. 2017. Batracofauna de los bosques de niebla y estribaciones del piedemonte en el municipio de Yopal,(Casanare), Orinoquia colombiana. *Biota Colombiana* 18:281-314.

Acosta-Galvis AR., Pinzón A. 2018. Una nueva rana nodriza (Anura: Dendrobatidae) de los bosques de niebla asociados a la cuenca del Orinoco de Colombia. *Biota Colombiana* 19(Sup. 1):160-190.

Acosta-Galvis AR., Vargas-Ramírez M. 2018. A new species of *Hyloxalus* Jiménez De La Espada, 1871 ‘‘1870’’(Anura: Dendrobatidae: Hyloxalinae) from a cloud forest near Bogotá, Colombia, with comments on the subpunctatus clade. *Vertebrate Zoology* 68:123-141.

Acosta-Galvis AR., Huertas-Salgado C., Rada M. 2006. Aproximación al conocimiento de los anfibios en una localidad del Magdalena medio (Departamento de Caldas, Colombia). *Revista de la Academia Colombiana de Ciencias Exactas, Físicas y Naturales* 30:291-303.

Acosta-Galvis AR., Rueda-Almonacid JV., Velásquez-Álvarez ÁA., Sánchez-Pacheco SJ., Peña-Prieto JA. 2006. Descubrimiento de una nueva especie de *Atelopus* (Bufonidae) para Colombia:¿Una luz de esperanza o el ocaso de los sapos Arlequines?. *Revista de la Academia Colombiana de Ciencias Exactas, Físicas y Naturales* 30:279-290.

Amezquita A, Marquez R, Medina R, Mejia-Vargas D, Kahn TR., Suarez G, Mazariegos L. 2013. A new species of Andean poison frog, *Andinobates* (Anura: Dendrobatidae), from the northwestern Andes of Colombia. *Zootaxa* 3620:163-178.

Anganoy-Criollo M. 2012. A new species of *Allobates* (Anura, Dendrobatidae) from the western flank of the Serranía de Perijá, Colombia. *Zootaxa* 3308:49-62.

Anganoy-Criollo M. Ramírez JP. 2017. New records of *Pristimantis carranguerorum* (Anura: Craugastoridae) from the Cordillera Oriental of Colombia. *Check List* 13:2138

Anganoy-Criollo MA. 2013. Tadpoles of high-Andean *Hyloxalus subpunctatus* (Anura: Dendrobatidae) with description of the larval variation and species distinction by larval morphology. *Papéis Avulsos de Zoologia* 53:211-224

Ardila-Robayo MC. 1999. Una nueva especie de *Atelopus* AMC Duméril & Bibron 1841 (Amphibia: Anura: Bufonidae) de la Cordillera Oriental de Colombia. *Revista de la Academia Colombiana de Ciencias Exactas, Físicas y Naturales* 23:139-142.

Ardila-Robayo MC., Ruiz-Carranza P.M. 1993. Una nueva especie de *Hyla* del grupo *larinopygion* (Amphibia: Anura: Hylidae) del sur de la Cordillera Central de Colombia. *Revista de la Academia Colombiana de Ciencias Exactas, Físicas y Naturales* 18:559-566.

Ardila-Robayo MC., Ruiz-Carranza PM. 1998. Una Nueva especie de *Atelopus* AMC Duméril & Bibron 1841 (Amphibia: Bufonidae) de la Cordillera Central Colombiana. *Revista de la Academia Colombiana de Ciencias Exactas, Físicas y Naturales* 22:281-285.

Ardila-Robayo MC., Acosta-Galvis A., Coloma L. 1999. Una nueva especie de *Colostethus* Cope, 1867 (Amphibia: Anura: Dendrobatidae) de la Cordillera Oriental colombiana. *Revista de la Academia Colombiana de Ciencias Exactas, Físicas y Naturales* 23:239-244.

Arenas-Rodríguez A., Vargas JFR., Hoyos JM. 2018. Comparative description and ossification patterns of *Dendropsophus labialis* (Peters, 1863) and *Scinax ruber* (Laurenti, 1758)(Anura: Hylidae). *PeerJ* 6:e4525.

Barrio-Amorós CL, 2006. A new species of Phyllomedusa (Anura: Hylidae: Phyllomedusinae) from northern Venezuela. *Zootaxa* 1309: 55-68.

Barrio-Amorós CL, Santos JC. 2012. A phylogeny for *Aromobates* (Anura: Dendrobatidae) with description of three new species from the Andes of Venezuela, taxonomic comments on *Aromobates saltuensis*, *A. inflexus*, and notes on the conservation status of the genus. *Zootaxa* 3422:1-31.

Barrio-Amorós CL, Díaz A, Mueses-Cisneros JJ., Infante E, Chacon A. 2006. *Hyla vigilans* Solano, 1971, a second species for the genus *Scarthyla*, redescription and distribution in Venezuela and Colombia. *Zootaxa* 1349:1-18.

Barrio-Amorós CL, Orellana A, Chacón A. (2004). A new species of *Scinax* (Anura: Hylidae) from the Andes of Venezuela. *Journal of Herpetology* 38:105-112.

Bernal MH, Páez CA. 2005. Composición y distribución de los anfibios de la cuenca del río Coello (Tolima), Colombia. *Actualidades Biológicas* 27:87-92.

Bolívar-García W., Giraldo A., Mendez J. 2011. Amphibia, Anura, Strabomantidae, *Pristimantis palmeri* Boulenger, 1912: Distribution extension for the Central Cordillera, Colombia. *Check List* 7:9-10.

Bolívar-García W., Grant T., Osorio L.A. 1999. Combat behavior in *Centrolene buckleyi* and other centrolenid frogs. *Alytes* 16:77-83.

Boulenger GA. 1896. II.—Descriptions of new Reptiles and Batrachians from Colombia. *Journal of Natural History* 17:16-21.

Boulenger GA. 1899. XLII.—Descriptions of new Batrachians in the collection of the British Museum (Natural History). Journal of Natural History, 3:273-277.

Boulenger GA. 1908. LXX.—Descriptions of new batrachians and reptiles discovered by Mr. MG Palmer in South-western Colombia. *Journal of Natural History* 2:515-522.

Boulenger GA. 1912. XIX.—Descriptions of new Batrachians from the Andes of South America, preserved in the British Museum. *Journal of Natural History* 10:185-191.

Bravo-Valencia L, Rivera-Correa M. 2011. A new species of harlequin frog (Bufonidae: *Atelopus*) with an unusual behavior from Andes of Colombia. *Zootaxa* 3045:57-67.

Buitrago-González W, López-Guzmán JH,Vargas-Salinas F. 2016. *Niceforonia adenobrachia* Ardila-Robayo, Ruiz-Carranza & Barrera-Rodríguez, 1996 (Amphibia: Anura: Craugastoridae): extension of geographical distribution in the Central Andes of Colombia. *Check List* 12:1845.

Cannatella DC. 1980. A review of the Phyllomedusa buckleyi group (Anura: Hylidae). *Occasional Papers of the Museum of Natural History, University of Kansas* 87:1-40.

Cannatella DC. 1981. A new *Atelopus* from Ecuador and Colombia. *Journal of Herpetology* 15:133-138.

Cannatella DC, Duellman WE. 1984. Leptodactylid frogs of the *Physalaemus pustulosus* group. *Copeia 4:*902-921.

Castro-Herrera F, Vargas-Salinas F. 2008. Anfibios y reptiles en el departamento del Valle del Cauca, Colombia. *Biota Colombiana* 9:251-277.

Cisneros-Heredia DF., Mcdiarmid RW. 2007. Revision of the characters of Centrolenidae (Amphibia: Anura: Athesphatanura), with comments on its taxonomy and the description of new taxa of glassfrogs. *Zootaxa* 1572: 1 82.

Cisneros-Heredia DF, Gluesenkamp A.G. 2010. A new Andean toad of the genus *Osornophryne* (Amphibia: Anura: Bufonidae) from northwestern Ecuador, with taxonomic remarks on the genus. *Avances en Ciencias e Ingenierías* 2:B64-B73.

Cochran DM, Goin CJ. 1970. Frogs of Colombia. *Bulletin of the United States National Museum*:1–655. DOI: 10.5479/si.03629236.288.1.

Colección de Herpetología de la Universidad del Valle. 2016. Colección de anfibios y reptiles del Laboratorio de Herpetología de la Universidad del Valle (UV-C).

Coloma LA, Duellman WE, Almendáriz A, Ron SR, Terán Valdez A, Guayasamin JM. 2010. Five new (extinct?) species of *Atelopus* (Anura: Bufonidae) from Andean Colombia, Ecuador, and Peru. Zootaxa 2574:1-54.

Daza JM, Barrientos LS. 2005. Geographic distribution: *Cochranella daidalea* *Herpetological Review* 36:198-199.

Duarte-Marín S, González-Acosta C, Vargas-Salinas F. 2018. Estructura y composición de ensamblajes de anfibios en tres tipos de hábitat en el Parque Nacional Natural Selva de Florencia, Cordillera Central de Colombia. *Revista de la Academia Colombiana de Ciencias Exactas, Físicas y Naturales* 42:227-236.

Duellman WE. 1971. A Taxonomic Review of South American Hylid Frogs: Genus *Phrynohyas*. *Occasional Papers of the Museum of Natural History of the University of Kansas* 4:1-21.

Duellman WE. 1972. A review of the Neotropical frogs of the *Hyla bogotensis* group. *Occasional Papers of the Museum of Natural History, University of Kansas* 11:1-31.

Duellman WE. 1972a. South American frogs of the *Hyla rostrata* group (Amphibia, Anura, Hylidae). *Zoologische Mededelingen* 47:177-192.

Duellman WE. 1973. Descriptions of new hylid frogs from Colombia and Ecuador. *Herpetologica* 29: 219-227.

Duellman WE. 1974. Taxonomic notes on *Phyllomedusa* (Anura: Hylidae) from the upper Amazon basin. *Herpetologica* 30: 105-112.

Duellman WE. 1980. The identity of *Centrolenella grandisonae* Cochran and Goin (Anura: Centrolenidae). *Transactions of the Kansas Academy of Science* 1:26-32.

Duellman WE. 1981. Three new species of centrolenid frogs from the Pacific versant of Ecuador and Colombia. *Occasional Papers of the Museum of Natural History of the University of Kansas* 88:1-9.

Duellman WE. 1983. A new species of marsupial frog (Hylidae: *Gastrotheca*) from Colombia and Ecuador. *Copeia* 4: 868-874.

Duellman WE. 1987. The taxonomic status of populations of hylid marsupial frogs referred to *Gastrotheca argenteovirens* (Boettger). *Journal of herpetology* 21:38-47.

Duellman WE. 1989. New species of hylid frogs from the Andes of Colombia and Venezuela. *Occasional Papers of the Museum of Natural History, University of Kansas* 131:1-12.

Duellman WE, Altig R. 1978. New species of tree frogs (family Hylidae) from the Andes of Colombia and Ecuador. *Herpetologica* 34:177-185.

Duellman WE, Berger TJ. 1982. A new species of Andean treefrog (Hylidae). *Herpetologica* 38:456-460.

Duellman WE, Burrowes PA. 1986. A new species of marsupial frog (Hylidae: *Gastrotheca*) from the Andes of southern Colombia. *Occasional Papers of the Museum of Natural History, University of Kansas* 120:1-11.

Duellman WE, Burrowes PA. 1989. New species of frogs, *Centrolenella*, from the Pacific versant of Ecuador and southern Colombia. *Occasional Papers of the Museum of Natural History of the University of Kansas* 59:1-14.

Duellman WE, Crump ML. 1974. Speciation in frogs of the *Hyla parviceps* group in the upper Amazon Basin. *Occasional Papers of the Museum of Natural History, University of Kansas* 23:1-40.

Duellman WE, Fouquette Jr MJ. 1968. Middle American frogs of the *Hyla microcephala* group. Ranas centroamericanas del grupo *Hyla microcephala*. *The University of Kansas Publications, Museum of Natural History* 17:517-557.

Duellman WE, Hillis DM. 1987. Marsupial frogs (Anura: Hylidae: *Gastrotheca*) of the Ecuadorian Andes: resolution of taxonomic problems and phylogenetic relationships. *Herpetologica* 43:141-173.

Duellman WE, Hillis DM. 1990. Systematics of frogs of the *Hyla larinopygion* group. *Occasional Papers of the Museum of Natural History, The University of Kansas* 134:1-23.

Duellman WE, Pyles RA. 1980. A new marsupial frog (Hylidae: *Gastrotheca*) from the Andes of Ecuador. *Occasional Papers of the Museum of Natural History, University of Kansas* 84: 1-13.

Duellman WE, Simmons JE. 1977. A new species of *Eleutherodactylus* (Anura: Leptodactylidae) from the Cordillera Oriental of Colombia. *Proceedings of the Biological Society of Washington* 90:60-65.

Duellman WE, Trueb L. 1983. Frogs of the *Hyla columbiana* group: taxonomy and phylogenetic relationships. Pp. 33-51. in A.G.J. Rhodin and K. Miyata (Eds.). Advances in herpetology and evolutionary biology. Museum of Comparative Zoology, Harvard University, Cambridge, MA.

Echavarría-Rentería JD, Copete-Mosquera LA, Rengifo-Mosquera JT, Pino-Mosquera YL, Rengifo-Palacios MY, Abadia-Bonilla D. 2015. Primer reporte de *Dendropsophus columbianus* (Boettger, 1892)(Anura: Hylidae) para el departamento del Chocó, Colombia. *Revista Institucional Universidad Tecnológica del Chocó Investigación Biodiversidad y Desarrollo* 34:47-50.

Enciso-Calle MP, Viuche-Lozano A, Anganoy-Criollo M, Bernal MH. 2017. Rediscovery of *Atelopus subornatus* Werner, 1899 (Anura: Bufonidae), with a redescription of the tadpole. *Zootaxa* 4344:160-162.

Faivovich J, Moravec J, Cisneros-Heredia DF, Köhler J. 2006. A new species of the *Hypsiboas benitezi* group from the western Amazon basin (Amphibia: Anura: Hylidae). *Herpetologica* 62:96-108.

Fernández-Roldán JD. 2014. *Pristimantis nervicus*. *Catálogo de Anfibios y Reptiles de Colombia*. 2: 25-29.

Frost DR. 2017. Amphibian Species of the World: an Online Reference. Version 6.0 (10 december 2017). Accesible at: http://research.amnh.org/herpetology/amphibia/index.html.

Frost DR. 2018. Amphibian Species of the World: an Online Reference. Version 6.0 (10 january 2018). Accesible at: http://research.amnh.org/herpetology/amphibia/index.html.

Gaige HT. 1933. A new *Gastrotheca* from Colombia. *Occasional Papers of the Museum of Zoology, University of Michigan* 263:1–3.

Galeano SP, Urbina JC. 2004. Geographic distribution: *Eleutherodactylus suetus*. *Herpetological Review* 35:281.

Garcia JC, Lynch JD. 2006. A new species of frog (genus Eleutherodactylus) from a cloud forest in Western Colombia. Zootaxa 1171: 39-45.

Goin CJ, Cochran DM. 1963. Two new genera of leptodactylid frogs from Colombia. P*roceedings of the California Academy of Sciences* 31:499 505.

Gonzáles Durán GA, Targino M, Rada M, Grant T. 2017 Phylogenetic relationships and morphology of the *Pristimantis leptolophus* species group (Amphibia: Anura: Brachycephaloidea), with the recognition of a new species group in *Pristimantis* Jiménez de la Espada, 1870. *Zootaxa* 4243:42-74

Gonzáles-Durán GA. 2016. A new small frog species of the genus *Pristimantis* (Anura: Craugastoridae) from the northern paramos of Colombia. *Zootaxa* 4066:421-437.

Grant T. 1999. Una nueva especie de *Rhamphophryne* (Anura: Bufonidae) de la Cordillera Central de Colombia. *Revista Academia Colombiana Ciencias Exactas, Físicas y Naturales* 23(Suplemento especial):287-299.

Grant T. 2004. On the identities of *Colostethus inguinalis* (Cope, 1868) and *C. panamensis* (Dunn, 1933), with comments on *C. latinasus* (Cope, 1863) (Anura: Dendrobatidae). *American Museum Novitates* 2004:1-24.

Grant T. 2007. A new, toxic species of *Colostethus* (Anura: Dendrobatidae: Colostethinae) from the Cordillera Central of Colombia. *Zootaxa* 1555:39-51.

Grant T, Acosta AR, Rada M. 2007. A name for the species of *Allobates* (Anura: Dendrobatoidea: Aromobatidae) from the Magdalena Valley of Colombia. *Copeia* 4:844-854.

Grant T., Ardila-Robayo MC. 2002. A new species of Colostethus (Anura: Dendrobatidae) from the eastern slopes of the Cordillera Oriental of Colombia. *Herpetologica* 58:252-260.

Grant T., Bolivar GW. 2014. A new species of semiarboreal toad with a salamander like ear (Anura: Bufonidae: *Rhinella*). *Herpetologica* 70:198-210.

Grant T, Castro-Herrera F. 1998. The cloud forest *Colostethus* (Anura, Dendrobatidae) of a region of the Cordillera Occidental of Colombia. *Journal of Herpetology* 32:378-392.

Grant T., Frost DR., Caldwell JP, Gagliardo R, Haddad CFB, Kok PJR, Means BD, Noonan BP, Schargel W, Wheeler WC. 2006. Phylogenetic systematics of dart-poison frogs and their relatives(Anura: Athesphatanura: Dendrobatidae). *Bulletin ofthe American Museum of Natural History* 299:1-262.

Grant T., Humphrey EC, Myers CW. 1997. The median lingual process of frogs: a bizarre character of Old World ranoids discovered in South American dendrobatids. *American Museum Novitates* 1997:1-40.

Grant T., Myers CW. 2013. Review of the Frog Genus *Silverstoneia*,with Descriptions of Five New Species from the Colombian Chocó (Dendrobatidae: Colostethinae). *American Museum Novitates* 2013:1-58.

Grant T, Rada M, Anganoy-Criollo M, Batista A, Dias PH, Jeckel AM, Machado DJ, Rueda-Almonacid JV. 2017. Phylogenetic Systematics of Dart-Poison Frogs and their Relatives Revisited (Anura: Dendrobatoidea). *South American Journal of Herpetology* 12(Special Issue 1):S1–S90.

Grant T, Rodríguez LO. 2001. Two new species of frogs of the genus *Colostethus* (Dendrobatidae) from Peru and a redescription of *C. trilineatus* (Boulenger, 1883). *American Museum Novitates* 2001:1 24.

Guarnizo CE, Escallón C, Cannatella DC, Amézquita A. 2012. Congruence between acoustic traits and genealogical history reveals a new species of *Dendropsophus* (Anura: Hylidae) in the high Andes of Colombia. *Herpetologica* 68: 523–540.

Guayasamin JM., Krynak T, Krynak K, Culebras J, Hutter C. R.. 2015. Phenotypic plasticity raises questions for taxonomically important traits: a remarkable new Andean rainfrog (*Pristimantis*) with the ability to change skin texture. Z*oological Journal of the Linnean Society* 173:913-928.

Gutierrez-Cárdenas PDA. 2005. *Cochranella susatamai* (Susatama’s Glass Frog). H*erpetological Review* 36:73.

Gutiérrez-Cárdenas PDA, Rojas-Rivera A, Rivera-Correa M. 2013. *Dendropsophus norandinus* (north Andean treefrog). Diet. *Herpetological Review* 44:120-121.

Henández-Córdoba OD, Cardona-Botero VE, Castro-Herrera F. 2014. Amphibia, Anura, Bufonidae, *Atelopus eusebianus* (Rivero & Granados-Díaz, 1993): Distribution extension for Valle del Cauca, Colombia. *Check List* 10:682-683.

Heyer WR. 1978. Systematics of the *fuscus* group of frogs genus *Leptodactylus* (Amphibia: Leptodactylidae). Natural History Museum of Los Angeles County Science Bulletin, Los Angeles.

Heyer WR, De Sá RO. 2011. Variation, Systematics, and Relationships of the *Leptodactylus bolivianus* Complex (Amphibia: Anura: Leptodactylidae). S*mithsonian Contributions to Zoology* 635: 1-58.

Heyer WR. 1973. Systematics of the *marmoratus* group of the frog genus *Leptodactylus* (Amphibia, Leptodactylidae). *Natural History Museum of Los Angeles County - Contributions in Science* 251:1-50.

Heyer WR. 1979. Systematics of the *pentadactylus* species group of the frog genus *Leptodactylus* (Amphibia: Leptodactylidae). Smithsonian Institution Press, Washington.

Heyer WR. 1994. Variation within the *Leptodactylus podicipinus-wagneri* complex of frogs (Amphibia: Leptodactylidae). SSmithsonian Institution Press, Washington.

Heyer WR. 1997. Geographic variation in the frog genus *Vanzolinius* (Anura: Leptodactylidae). *Proceedings of the Biological Society of Washington* 110:338-365.

Heyer WR. 2005. Variation and taxonomic clarification of the large species of the *Leptodactylus pentadactylus* species group (Amphibia: Leptodactylidae) from Middle America, Northern South America, and Amazonia. *Arquivos de Zoologia* 37:269-348.

Hill RL., Martin KG, Stanley EL, Mendelson JR. 2018. A taxonomic review of the genus *Hemiphractus* (Anura: Hemiphractidae) in Panama: Description of Two New Species, Resurrection of *Hemiphractus panamensis* (Stejneger, 1917), and discussion of *Hemiphractus fasciatus* Peters, 1862 . *Zootaxa* 4429:495–512.

Hillis DM, de Sá R. 1988. Phylogeny and Taxonomy of the *Rana palmipes* Group (Salientia: Ranidae) *Herpetological Monographies* 2:1-26.

ICN. 2004. Colecciones científicas en línea.

IAvH. 2018. Colección de anfibios del Instituto Alexander von Humboldt.

Jaramillo Martinez AF, Valencia ZA, Cardona VE, Castro Herrera F, Cisneros Heredia DF. 2015. Range extension of *Cochranella mache* Guayasamin and Bonaccorso, 2004 (Anura: Centrolenidae) with comments on the distribution of *C. euknemos* (Savage and Starrett, 1967) in Colombia. *Herpetology Notes* 8:161-163.

Jerez A, Arroyo S, Ramirez-Pinilla MP. 2001. *Eleutherodactylus lutitus*. Geographic distribution. *Herpetological Review* 34:270.

Jiménez Rivillas C, Vargas LM, Fang, JM, Di Filippo J, Daza JM. 2013. Advertisement Call of *Diasporus anthrax* (Lynch, 2001) (Anura: Eleutherodactylidae) with Comparisons to Calls from Congeneric Species. *South American Journal of Herpetology* 8:1-4

Jungfer KH, Faivovich J, Padial JM, Castroviejo-Fisher S, Lyra ML, von MB, …, Haddad CFB. 2013. Systematics of spiny-backed treefrogs (Hylidae: *Osteocephalus*): an Amazonian puzzle. *Zoologica Scripta* 42:351–380.

Kaplan M. 1991. A new species of *Hyla* from the eastern slope of the Cordillera Oriental in northern Colombia. *Journal of Herpetology* 25:313-316.

Kaplan M. 1994. A new species of frog of the genus *Hyla* from the Cordillera Oriental in northern Colombia with comments on the taxonomy of *Hyla minuta*. *Journal of Herpetology* 28:79-87.

Kaplan M. 1997. On the status of *Hyla bogerti* Cochran and Goin. *Journal of Herpetology* 31:536-541.

Kaplan M, Ruiz-Carranza PM. 1997. Two new species of *Hyla* from the Andes of central Colombia and their relationships to other small Andean *Hyla*. *Journal of Herpetology* 31:230-244.

Kattan G. 1986. Nueva especie de rana (*Atelopus*) de los Farallones de Cali, Cordillera Occidental de Colombia. *Caldasia* 14:651-657.

Kluge AG. 1979. The gladiator frogs of Middle america and Colombia-A -reevaluation Of their systematics (Anura: Hylidae). *Occasional Papers of the Museum of Zoology University of Michigan* 688:1-24.

La Marca E, Garcia Perez JE, Renjifo JM. 1990. Una nueva especie de *Atelopus* (Amphibia: Anura: Bufonidae) del Paramo de Tama, Estado Apure, Venezuela. *Caldasia* 16:97-104.

Lynch JD. 1973. The systematic status of the Colombian leptodactylid frog, *Leptodactylus mantipus* Boulenger. *Herpetologica* 29:232–235.

Lynch JD. 1975. A review of the broad headed eleutherodactyline frogs of South America (Leptodactylidae). *Occasional Papers of the Museum of Natural History, University of Kansas* 38:1-46.

Lynch JD. 1975a. The identity of the frog *Eleutherodactylus conspicillatus* (Günther), with descriptions of two related species from northwestern South America (Amphibia, Leptodactylidae). C*ontributions in Science. Natural History Museum of Los Angeles County* 272:1–19.

Lynch JD. 1976. A new high Andean slope species of *Eleutherodactylus* (Amphibia: Leptodactylidae) from Colombia and Ecuador. *Proceedings of the Biological Society of Washington* 88:351–354.

Lynch JD. 1976a. Three new leptodactylid frogs (genus *Eleutherodactylus*) from the Andean slopes of Colombia and Ecuador. *Herpetologica* 32:310–317.

Lynch JD. 1977. A new frog (Leptodactylidae: *Eleutherodactylus*) from the Pacific lowlands of Ecuador. *Copeia* 1977:282–284.

Lynch JD. 1977a. A new species of *Eleutherodactylus* from the Cordillera Occidental of Colombia (Amphibia: Anura: Leptodactylidae). *Occasional Papers of the Museum of Zoology, University of Michigan* 678:1–6.

Lynch JD. 1978. A new eleutherodactyline frog from the Andes of northern Colombia. *Copeia* 1978:17–21.

Lynch JD. 1980. A taxonomic and distributional synopsis of the Amazonian frogs of the genus *Eleutherodactylus*. *American Museum Novitates* 2696:1–24.

Lynch JD. 1980a. New species of *Eleutherodactylus* of Colombia (Amphibia: Leptodactylidae). I: Five new species from the paramos of the Cordillera Central. *Caldasia* 13:165–188.

Lynch JD. 1980b. Systematic status and distribution of some poorly known frogs of the genus *Eleutherodactylus* from the Chocoan lowlands of South America. *Herpetologica* 36:175–189.

Lynch JD. 1981. Leptodactylid frogs of the genus *Eleutherodactylus* in the Andes of northern Ecuador and adjacent Colombia. *Miscellaneous Publication. Museum of Natural History, University of Kansas* 72:1–46.

Lynch JD. 1981a. The systematic status of *Amblyphrynus ingeri* (Amphibia: Leptodactylidae) with the description of an allied species in western Colombia. *Caldasia* 13:313–332.

Lynch JD. 1981b. Two new species of *Eleutherodactylus* from western Colombia (Amphibia: Anura: Leptodactylidae). *Occasional Papers of the Museum of Zoology, University of Michigan* 697:1–12.

Lynch JD. 1982. Two new species of poison-dart frogs (*Colostethus*) from Colombia. *Herpetologica* 38:366-374.

Lynch JD. 1983. A new leptodactylid frog from the Cordillera Oriental of Colombia. Pp. 52-57. Rhodin, A. G. J., and K. Miyata (eds.). Advances in Herpetology and Evolutionary Biology. Essays in Honor of Ernest E. Williams. Museum of Comparative Zoology, Harvard University, Cambridge, MA.

Lynch JD. 1984. A new species of Eleutherodactylus (Amphibia: Anura: Leptodactylidae) from southern Andean Colombia. *Herpetologica* 40:234–237.

Lynch JD. 1984a. New frogs (Leptodactylidae: *Eleutherodactylus*) from cloud forest of the northern Cordillera Oriental, Colombia. *Contributions in Biology and Geology. Milwaukee Public Museum* 60:1–19.

Lynch JD. 1985. Mimetic and non-mimetc populations of *Eleutherodactylus gaigeae* (Dunn) in lower Central America and Colombia (Amphibia: Anura: Leptodactylidae). S*tudies on Neotropical Fauna and Environment* 20:195-202.

Lynch JD. 1986. New species of *Eleutherodactylus* of Colombia (Amphibia: Leptodactylidae) II: Four species from the cloud forests of the western Cordilleras. *Caldasia* 15:629–647.

Lynch JD. 1989. A review of the leptodactylid frogs of the genus *Pseudopaludicola* in Northern South America. *Copeia* 1989:577-588.

Lynch JD. 1989a. Intrageneric relationships of mainland *Eleutherodactylus* (Leptodactylidae). I. A review of the frogs assigned to the *Eleutherodactylus discoidalis* species group. *Contributions in Biology and Geology. Milwaukee Public Museum* 79:1-25.

Lynch JD. 1990. A new large species of streamside *Eleutherodactylus* from western Colombia (Amphibia: Leptodactylidae). *Herpetologica* 46:135–142.

Lynch JD. 1990a. A new ocellated frog (Centrolenidae) from western Colombia. *Proceedings of the Biological Society of Washington* 103:35–38.

Lynch JD. 1991. New diminutive *Eleutherodactylus* from the Cordillera Central of Colombia (Amphibia: Leptodactylidae). *Journal of Herpetology* 25:344–352.

Lynch JD. 1992. A new species of *Eleutherodactylus* (Amphibia: Leptodactylidae) from western Colombia with cranial co-ossification. *Copeia* 1992:826–831.

Lynch JD. 1992a. A new species of leptodactylid frog (*Eleutherodactylus*) from southwestern Colombia. *Herpetologica* 48:347–350.

Lynch JD. 1992b. Distribution and variation in a Colombian frog, *Eleutherodactylus erythropleura* (Amphibia: Leptodactylidae). *Studies on Neotropical Fauna and Environment* 27:211-226.

Lynch JD. 1992c. Two new species of *Eleutherodactylus* from southwestern Colombia and the proposal of a new species group (Amphibia: Leptodactylidae). *Journal of Herpetology* 26:53–59.

Lynch JD. 1993. A new harlequin frog from the Cordillera Oriental of Colombia (Anura: Bufonidae: *Atelopus*). *Alytes* 11:77-87.

Lynch JD. 1993a. A new centrolenid frog from the Andes of western Colombia. *Revista de la Academia Colombiana de Ciencias Exactas, Físicas y Naturales* 18:567-569.

Lynch JD. 1994. A new species of frog (genus *Eleutherodactylus*: Leptodactylidae) from a cloud forest in Departamento de Santander, Colombia. *Revista de la Academia Colombiana de Ciencias Exactas, Físicas y Naturales* 19:205–208.

Lynch JD. 1994a. A new species of high-altitude frog (*Eleutherodactylus*: Leptodactylidae) from the Cordillera Oriental of Colombia. *Revista de la Academia Colombiana de Ciencias Exactas, Físicas y Naturales* 19:195-203.

Lynch JD. 1994b. Two new species of the *Eleutherodactylus conspicillatus* group (Amphibia: Leptodactylidae) from the Cordillera Oriental of Colombia. *Revista de la Academia Colombiana de Ciencias Exactas, Físicas y Naturales* 19:187–193.

Lynch JD. 1995. Three new species of *Eleutherodactylus* (Amphibia: Leptodactylidae) from paramos of the Cordillera Occidental of Colombia. J*ournal of Herpetology* 29:513-521.

Lynch JD. 1996. New frog (Eleutherodactylus: Leptodactylidae) from the Andes of eastern Colombia, part of a remarkable pattern of distribution. *Copeia* 1996:103–108.

Lynch JD. 1996a. New frogs of the genus *Eleutherodactylus* (family Leptodactylidae) from the San Antonio region of the Colombian Cordillera Occidental. *Revista de la Academia Colombiana de Ciencias Exactas, Físicas y Naturales* 20:331–345.

Lynch JD. 1997. Intrageneric relationships of mainland *Eleutherodactylus* II. A review of the *Eleutherodactylus sulcatus* group. *Revista de la Academia Colombiana de Ciencias Exactas, Físicas y Naturales* 21:353–372.

Lynch JD. 1998. A new frog (genus *Eleutherodactylus*) from cloud forests of southern Boyaca. *Revista de la Academia Colombiana de Ciencias Exactas, Físicas y Naturales* 22:429-432.

Lynch JD. 1998a. New Species of *Eleutherodactylus* from The Cordillera Occidental of western Colombia with synopsis of the distribution of species in Western Colombia. R*evista de la Academia Colombiana de Ciencias Exactas, Físicas y Naturales* 22:117-148.

Lynch JD. 1999. Lista anotada y clave para las ranas (género *Eleutherodactylus*) chocoanas del valle Del cauca, y apuntes sobre las especies de la cordillera occidental adyacente. *Caldasia* 21:184-202.

Lynch JD. 2000. A new species of frog, genus *Eleutherodactylus* (Leptodactylidae), from the Sabana de Bogotá. *Revista de la Academia Colombiana de Ciencias Exactas, Físicas y Naturales* 24:435–439.

Lynch JD. 2001. A small amphibian fauna from a previously unexplored Paramo of the Cordillera Occidental in western Colombia. *Journal of Herpetology* 35:221-231.

Lynch JD. 2001a. Three new rainfrogs of the *Eleutherodactylus diastema* group from Colombia and Panama. *Revista de la Academia Colombiana de Ciencias Exactas, Físicas y Naturales* 2S:287-297.

Lynch JD. 2003. A new species of frog from northeastern Colombia (genus *Eleutherodactylus*: Leptodactylidae). *Revista de la Academia Colombiana de Ciencias Exactas, Físicas y Naturales* 27:287-289.

Lynch JD. 2003a. New species of frogs (*Eleutherodactylus*: Leptodactylidae) from the Cordillera Oriental of Norte de Santander and Santander, Colombia. *Revista de la Academia Colombiana de Ciencias Exactas, Físicas y Naturales* 27:449-460.

Lynch JD. 2003b. Two new frogs (*Eleutherodactylus*) from the Serrania de Perijá, Colombia. *Revista de la Academia Colombiana de Ciencias Exactas, Físicas y Naturales* 27:613-617.

Lynch JD. 2005. Discovery of the richest frog fauna in the World—an exploration of the forests to the north of Leticia. *Revista de la Academia Colombiana de Ciencias Exactas, Físicas y Naturales* 29:581-588.

Lynch JD. 2006. The amphibian fauna in the Villavicencio region of eastern Colombia. *Caldasia* 28:135-155.

Lynch JD. 2008. A taxonomic revision of frogs of the genus *Cryptobatrachus* (Anura: Hemiphractidae). *Zootaxa* 1883:28-68.

Lynch JD., Ardila-Robayo MC. 1999. The *Eleutherodactylus* of the *taeniatus* complex in western Colombia: taxonomy and distribution. R*evista de la Academia Colombiana de Ciencias Exactas, Físicas y Naturales* 23:615–624.

Lynch JD., Ardila-Robayo MC. 2004. A new Colombian frog of the genus *Eleutherodactylus* from the northern Cordillera Occidental. *Revista de la Academia Colombiana de Ciencias Exactas, Físicas y Naturales* 28:403–408.

Lynch JD, Burrowes PA. 1990. The frogs of the genus *Eleutherodactylus* (family Leptodactylidae) at the La Planada Reserve in southwestern Colombia with descriptions of eight new species. *Occasional Papers of the Museum of Natural History, University of Kansas* 136:1–31.

Lynch JD, Duellman WE. 1973. A review of the Centrolenid frogs of Ecuador, with descriptions of new species. *Occasional Papers of the Museum of Natural History of the University of Kansas* 16:1-66.

Lynch JD, Duellman WE. 1980. The *Eleutherodactylus* of the Amazonian slopes of the Ecuadorian Andes (Anura: Leptodactylidae). *Miscellaneous Publication. Museum of Natural History, University of Kansas* 69:1-86.

Lynch JD, Myers CW. 1983. Frogs of the *fitzingeri* group of *Eleutherodactylus* in eastern Panama and Chocoan South America (Leptodactylidae). *Bulletin of the American Museum of Natural History* 175:481-572.

Lynch JD, Renjifo JM. 1990. Two new toads (Bufonidae: *Rhamphophryne*) from the northern Andes of Colombia. *Journal of Herpetology* 24:364-371.

Lynch JD, Rueda-Almonacid JV. 1997. Three new frogs (*Eleutherodactylus*: Leptodactylidae) from cloud forests in eastern Departamento Caldas, Colombia. *Revista de la Academia Colombiana de Ciencias Exactas, Físicas y Naturales* 21:131–142.

Lynch JD, Rueda-Almonacid JV. 1998. Additional new species of frogs (genus *Eleutherodactylus*) from cloud forests of eastern Departamento de Caldas, Colombia. *Revista de la Academia Colombiana de Ciencias Exactas, Físicas y Naturales* 22:287–298.

Lynch JD, Rueda-Almonacid JV. 1998. New frogs of the genus *Eleutherodactylus* from the eastern flank of the northern Cordillera Central of Colombia. R*evista de la Academia Colombiana de Ciencias Exactas, Físicas y Naturales* 22:561-570.

Lynch JD, Rueda-Almonacid JV. 1999. New species of frogs from low and moderate elevations from the Caldas transect of the eastern flank of the Cordillera Central. *Revista de la Academia Colombiana de Ciencias Exactas, Físicas y Naturales* 23:307-314.

Lynch JD, Ruiz-Carranza PM. 1981. A new species of toad (Anura: Bufonidae) from the Cordillera Occidental southern Colombia. *Lozania: Acta zoologica colombiana* 33:1-7.

Lynch JD., Ruiz-Carranza PM. 1982. A new genus and species of poison dart frog (Amphibia: Dendrobatidae) from the Andes of northern Colombia. P*roceedings of the Biological Society of Washington* 95:557-562.

Lynch JD, Ruiz-Carranza PM. 1983. New frogs of the genus *Eleutherodactylus* from the Andes of southern Colombia. *Transactions of the Kansas Academy of Science* 86:99–112.

Lynch JD, Ruiz-Carranza PM. 1996. A remarkable new centrolenid frog from Colombia with a review of nuptial excrescences in the family. *Herpetologica* 52:525–535.

Lynch JD, Ruiz-Carranza PM. 1996. New sister species of *Eleutherodactylus* from the Cordillera Occidental of southwestern Colombia(Amphibia: Salientia: Leptodactylidae). *Revista de la Academia Colombiana de Ciencias Exactas, Físicas y Naturales* 20:347-363.

Lynch JD, Ruiz-Carranza PM, Ardila-Robayo MC. 1994. The identities of the Colombian frogs confused with *Eleutherodactylus latidiscus* (Boulenger) (Amphibia: Anura: Leptodactylidae). *Occasional Papers of the Museum of Natural History, University of Kansas* 170:1–42.

Lynch JD, Ruiz-Carranza PM, Ardila-Robayo MC.. 1996. Three new species of *Eleutherodactylus* (Amphibia: Leptodactylidae) from high elevations of the Cordillera Central of Colombia. *Caldasia* 18:329–342.

Lynch JD, Súarez-Mayorga AM. 2000. A new frog (*Eleutherodactylus*: Leptodactylidae) from the southern part of the Cordillera Oriental of Colombia. R*evista de la Academia Colombiana de Ciencias Exactas, Físicas y Naturales* 24:289-293.

Lynch JD, Suárez-Mayorga AM. 2001. The distributions of the gladiator frogs (*Hyla boans* group) in Colombia, with comments on size variation and sympatry. *Caldasia* 23:491-507.

Lynch JD, Súarez-Mayorga AM. 2003. Two additional new species of *Eleutherodactylus* (Leptodactylidae) from southwestern Colombia. *Revista de la Academia Colombiana de Ciencias Exactas, Físicas y Naturales* 27:607-612.

Lynch JD, Suárez-Mayorga AM. 2011. Clave ilustrada de los renacuajos en las tierras bajas al oriente de los Andes con énfasis en Hylidae. *Caldasia* 33:235-270.

Malambo LC, Madrid Ordóñez MA. 2008. Geographic distribution of *Limnophys sulcatus*,*Rhinella castaneotica* and *Scinax cruentommus* (Amphibia: Anura) for Colombia. *Revista de la Academia Colombiana de Ciencias Exactas, Físicas y Naturales* 32:285-289.

Malambo LC, González Ibarra JF, Gomez Polania YC. 2013. Amphibia, Anura, Centrolenidae *Teratohyla midas* (Lynch & Duellman, 1973) and *Cochranella resplendens* (Lynch & Duellman, 1973): First and second record respectively for Colombia. *Check List* 9:894–896.

Malambo LC, González Ibarra JF, Gomez Polania YC. 2017. Rediscovery of *Centrolene solitaria* (Anura: Centrolenidae) from Colombia. *Phyllomedusa* 16:97-99.

Marín-Castaño CM, Molina-Zuluaga C, Restrepo A. 2018. A new species of *Leucostethus* (Anura: Dendrobatidae) from the eastern versant of the Central Cordillera of Colombia and the phylogenetic status of *Colostethus fraterdanieli*. *Zootaxa* 4461:359–380.

Márquez R, Mejía-Vargas D, Palacios-Rodríguez P, Ramírez-Castañeda V, Amézquita A. 2017. A new species of *Andinobates* (Anura: Dendrobatidae) from the Urabá region of Colombia. Zootaxa. 4290:531–546.

Méndez-Narváez J, Ortiz-Navia JO, Bolívar-García W. 2014. *Hypsiboas pugnax* Schmidt, 1857 and *Scinax ruber* Laurenti, 1768 (Amphibia: Anura): Distribution extension in the Río Cauca Valley, Colombia. *Check List* 10:409–410.

Méndez-Narváez J, Ospina-Sarria JJ, Bolívar-García W. 2009. Amphibia, Anura, Leptodactylidae, *Leptodactylus fragilis*: Distribution extension, Colombia. *Check List* 5:460-46.

Meneses Pelayo E, Chinchilla Lemus W, Ramírez Pinilla MP. 2017 Filling gaps and update of the distribution of *Strabomantis ingeri* (Cochran & Goin, 1961) (Anura: Craugastoridae) in Colombia. *Check List* 13:17–20.

Meza-Joya FL. 2016. First records of two rain frogs, genus *Pristimantis* (Anura, Craugastoridae), for Colombia. *Check List* 12:1971

Molina Zuluaga C, Cano E, Restrepo A, Rada M, Daza JM. 2017 Out of Amazonia: the unexpected trans Andean distribution of *Cochranella resplendens* (Lynch and Duellman, 1978) (Anura: Centrolenidae). *Zootaxa* 4238:268–274.

Montezuma MF, Mueses-Cisneros JJ. 2009. Amphibia, Anura, Hylidae, *Hyloscirtus tigrinus*: Distribution extension, first department record, Cauca and Huila, Colombia. *Check List* 5:243-245.

Mueses Cisneros JJ. 2003. El género Osornophryne (Amphibia: Anura) en Colombia. Caldasia. 25 (2): 419 427.

Mueses Cisneros JJ. 2005. Crítica a la asignación de la categoría de amenaza de *Gastrotheca ruizi* (Amphibia: Anura: Hylidae). *Revista de la Academia Colombiana de Ciencias Exactas, Físicas y Naturales* 29:303-308.

Mueses Cisneros JJ. 2005a. Fauna Anfibia del Valle de Sibundoy, Putumayo Colombia. The Amphibian Fauna of the Valle de Sibundoy, Putumayo Colombia. *Caldasia* 27:229-242.

Mueses Cisneros JJ. 2007. Fauna anura asociada a un sistema de charcos dentro de bosque en el kilómetro 11 carretera Leticia -Tarapacá (Amazonas-Colombia). *Caldasia* 29:387–395.

Mueses Cisneros JJ. 2007. Two new species of the genus *Eleutherodactylus* (Anura: Brachycephalidae) from Valle de Sibundoy, Putumayo, Colombia. *Zootaxa* 1498:35-43.

Mueses Cisneros JJ. 2009. *Rhaebo haematiticus* (Cope 1862): Un complejo de especies. Con redescripción de *Rhaebo hypomelas* (Boulenger 1913) y descripción de una nueva especie. *Herpetotropicos* 5:29–47.

Mueses-Cisneros JJ, Anganoy-Criollo MA. 2008. Una nueva especie del grupo *Hyloscirtus larinopygion* (Amphibia: Anura: Hylidae) del suroccidente de Colombia. *Papéis Avulsos de Zoologia* 48:129-138.

Mueses-Cisneros JJ, Cepeda-Quilindo B, Moreno-Quintero V. 2008. Una nueva especie de *Epipedobates* (Anura: Dendrobatidae) del suroccidente de Colombia. *Papéis Avulsos de Zoologia* 48:1–10.

Mueses Cisneros JJ, Cisneros Heredia DF, McDiarmid RW. 2012. A new Amazonian species of *Rhaebo* (Anura: Bufonidae) with comments on *Rhaebo glaberrimus* (Gunther, 1869) and *Rhaebo guttatus* (Schneider, 1799). *Zootaxa* 3447:22-40.

Mueses-Cisneros JJ, Moreno-Quintero V. 2012. Fauna anfibia de la Reserva Natural Biotopo Selva Húmeda, Barbacoas, Nariño, Colombia. *Herpetotropicos* 7:39-54.

Mueses-Cisneros JJ, Perdomo-Castillo IV. 2011 *Hyloscirtus tigrinus* Mueses-Cisneros & Anganoy-Criollo, 2008: una especie amenazada, con comentarios sobre su distribución geográfica e historia natural. *Herpetotropicos* 5:93–103.

Mueses-Cisneros JJ, Perdomo-Castillo IV, Cepeda-Quilindo B. 2013. A new species of the genus *Pristimantis* (Anura: Craugastoridae) from southwestern Colombia. *Herpetotropicos* 9:37-45.

Muñoz-Arcos R, Guerrero-Cupacán JA, Cepeda-Quilindo B. 2016. First record of *Pristimantis crucifer* Boulenger, 1899 (Anura: Craugastoridae) from Colombia, Nariño Department. *Check List* 12:2021.

Myers CW. 1991. Distribution of the dendrobatid frog *Colostethus chocoensis* and description of a related species occurring macrosympatrically. *American Museum Novitates* 3010:1-15.

Myers CW, Burrowes PA. 1987. A new poison frog (*Dendrobates*) from Andean Colombia, with notes on a lowland relative. *American Museum Novitates* 2899:1-17.

Myers CW, Daly JW. 1976. Preliminary evaluation of skin toxins and vocalizations in taxonomic and evolutionary studies of poison-dart frogs (Dendrobatidae). *Bulletin of the American Museum of Natural History* 157:173–262.

Myers CW, Daly JW. 1980. Taxonomy and ecology of *Dendrobates bombetes*,a new Andean Poison frog with new skin toxins. *American Museum Novitates* 2692:1-23.

Myers CW, Duellman WE. 1982. A new species of *Hyla* from Cerro Colorado, and other tree frog records and geographical notes from western Panama. *American Museum Novitates* 2752:1-32.

Narvaes P, Trefaut Rodrigues M. 2009. Taxonomic revision of *Rhinella granulosa* species group (Amphibia,Anura, Bufonidae), with a description of a new species. *Arquivos de Zoologia* 40:1-73

Noble GK. 1920. Two new batrachians from Colombia. *Bulletin of the American Museum of Natural History* 42:441-446.

Ortega-Andrade HM. 2008. *Agalychnis spurrelli* Boulenger (Anura, Hylidae): variación, distribución y sinonimia. *Papéis Avulsos de Zoologia* 48:103-117.

Ortega Andrade HM, Venegas PJ. 2014. A new synonym for *Pristimantis luscombei* (Duellman and Mendelson 1995) and the description of a new species of *Pristimantis* from the upper Amazon basin (Amphibia: Craugastoridae). *Zootaxa* 3895:31–57 .

Osorio Dominguez D, Quintero Angel A. 2012. *Espadarana prosoblepon* Boettger, 1892 (Amphibia: Anura): Distribution extension on the western slopes of the Cordillera Central, Colombia. *Check List* 8:898–899.

Osorno Muñoz M, Ardila Robayo MC, Ruiz Carranza PM. 2001. Tres nuevas especie de *Atelopus* A. M. C. Dumeril & Bibron, 1841 (Amphibia: Bufonidae) de las partes altas de la Cordillera Oriental Colombiana. *Caldasia* 23:509-522.

Ospina Sarria JJ, Angarita Sierra T, Pedroza Banda R. 2015. A New Species of *Craugastor* (Anura: Craugastoridae) from the Magdalena River Valley, Colombia, with Evaluation of the Characters Used to Identify Species of the *Craugastor fitzingeri* Group. S*outh American Journal of Herpetology* 10:165-177.

Ospina-Sarria JJ, Mendez-Narvaez J, Burbano-Yandi C, Bolívar-GW. 2011. A new species of *Pristimantis* (Amphibia: Craugastoridae) with cranial crests from the Colombian Andes. *Zootaxa* 3111:37-48.

Ospina Sarria JJ, Velásques Trujillo DA, Bólivar García W. 2015. First records of the twoo poorly know Terrarana frogs *Pristimantis esmeraldas* (Guayasamin,2004) and *Strabomantis necerus* (Lynch, 1975) (Amphibia Anura Craugastoridae) for Colombia. *Herpetology Notes* 8:27-30.

Pérez-Villota JE, Anganoy-Criollo MA, Betancourth-Cundar M. 2009. Amphibia, Anura, Hylidae, *Nyctimantis rugiceps*: Distribution extension, Colombia. *Check List* 5:270-272.

Pramuk JB. 2006. Phylogeny of South American *Bufo* (Anura: Bufonidae) inferred from combined evidence. *Zoological Journal of the Linnean Society* 146:407–452.

Pyburn WF, Fouquette Jr MJ. 1971. A new striped treefrog from central Colombia. *Journal of Herpetology* 5:97-101.

Pyburn WF, Lynch JD. 1981. Two little-known species of E*leutherodactylus* (Amphibia: Leptodactylidae) from the Sierra de la Macarena, Colombia. *Proceedings of the Biological Society of Washington* 94:404–412.

Rada M, Guayasamin JM. 2008. Redescripción de *Cochranella megista* (Rivero, 1985) y ampliación de la distribución de nueve ranas de cristal (Anura: Centrolenidae) en Colombia. *Papeis Avulsos de Zoologia* 48:99 100.

Rada M, Jeckel AM, Caorsi VZ, Barrientos LS, Rivera-Correa M, Grant T. 2017. A Remarkable New White Eyed Glassfrog Species of *Sachatamia* from Colombia (Anura: Centrolenidae), with Comments on the Systematics of the Genus. *South American Journal of Herpetology* 12:157-173.

Rada M, Ospina Sarria JJ, Guayasamin JM. 2017 A Taxonomic Review of Tan Brown Glassfrogs (Anura: Centrolenidae), with the Description of a New Species from Southwestern Colombia. *South American Journal of Herpetology* 12:136-156.

Rada M, Rueda Almonacid JV, Velásquez Álvarez AA, Sánchez Pacheco SJ. 2007. Descripción de las larvas de dos centrolénidos (Anura: Centrolenidae) del noroccidente de la Cordillera Oriental, Colombia. *Papéis Avulsos de Zoologia* 47:259 272.

Ramírez-Chaves HE, Pisso-Florez GA, Liévano-Bonilla AF, Ayerbe-Quiñones F, Anganoy-Criollo MA, Noguera-Urbano EA. 2018. On the distribution of the endemic Boettger’s Colombian Treefrog, *Dendropsophus columbianus* (Anura: Hylidae) with distribution extension in southwestern Colombia. *Herpetology Notes* 11:49–58.

Ramos E, Meza-Joya FL, Hernández-Jaimes C. 2018. Distribution and Conservation Status of *Andinobates virolinensis* (Dendrobatidae), a threatened Andean Poison Frog Endemic to Colombia. *Herpetological Conservation and Biology* 13:58–69.

Restrepo A, Molina Zuluaga C, Hurtado JP, Marín CM, Daza JM. 2017. Amphibians and reptiles from two localities in the northern Andes of Colombia. *Check List* 13:203–237.

Rivera-Correa M. (2010). Amphibia, Centrolenidae, *Centrolene antioquiense* (Noble, 1920): New records and geographical distribution in Colombia. *Check List* 6:220-221.

Rivera-Correa M, Daza JM. 2016. Molecular phylogenetics of the *Pristimantis lacrimosus* species group (Anura: Craugastoridae) with the description of a new species from Colombia. *Acta Herpetologica* 11:31–45.

Rivera-Correa M, Duarte-Cubides F, Rueda-Almonacid JV, Daza JM. 2013. A new red-eyed treefrog of *Agalychnis* (Anura: Hylidae: Phyllomedusinae) from middle Magdalena River valley of Colombia with comments on its phylogenetic position. *Zootaxa* 3636:85–100.

Rivera-Correa M, Faivovich J. 2013. A New Species of *Hyloscirtus* (Anura: Hylidae) from Colombia, with a rediagnosis of *Hyloscirtus larinopygion* (Duellman, 1973). *Herpetologica* 69:298–313.

Rivera-Correa M, Gutiérrez-Cárdenas PDA. 2012. A new highland species of treefrog of the *Dendropsophus columbianus* group (Anura: Hylidae) from the Andes of Colombia. *Zootaxa* 3486: 50-62.

Rivera-Correa M. Jiménez-Rivillas C, Daza JM. 2017. Phylogenetic Analysis of the Neotropical *Pristimantis leptolophus* species Group (Anura: Craugastoridae): Molecular Approach and Description of A New Polymorphic Species. *Zootaxa* 4242:313–343

Rivera-Correa M, Lamadrid-Feris F, Crawford AJ. 2016. A new small golden frog of the genus *Pristimantis* (Anura: Craugastoridae) from an Andean cloud forest of Colombia. *Amphibia-Reptilia* 37:153–166.

Rivera-Correa M, Orrico VGD. 2013. Description and phylogenetic relationships of a new species of treefrog of the *Dendropsophus leucophyllatus* group (Anura: Hylidae) from Amazon basin of Colombia and with an exceptional color pattern. *Zootaxa* 3680:447-460.

Rivera-Prieto DA, Rivera-Correa M, Daza JMA. 2014 new colorful species of Pristimantis (Anura: Craugastoridae) from the eastern flank of the Cordillera Central in Colombia.Zootaxa 3900 (2): 223–242.

Rivero JA. 1963 Five new species of *Atelopus* from Colombia, with notes on other forms from Colombia and Ecuador. Caribbean Journal of Science 3:103–124.

Rivero JA. 1985. Nuevos centrolenidos de Colombia y Venezuela. *Brenesia* 23:335-373.

Rivero JA, Castaño C. 1990. A new and peculiar species of *Rhamphophryne* (Amphibia: Bufonidae) from Antioquia, Colombia. *Journal of Herpetology* 1:1 5.

Rivero JA, Granados-Díaz H. 1990 "1989". Nuevos Colostethus (Amphibia, Dendrobatidae) del Departamento de Cauca, Colombia. Caribbean Journal of Science 25: 148–152.

Rivero JA, Serna MA. 1986. Dos nuevas especies de *Colostethus* (Amphibia, Dendrobatidae). *Caldasia* 15:525-531.

Rivero JA, Serna MA. 1988. Tres nuevas especies de *Eleutherodactylus* (Amphibia, Leptodactylidae) de Antioquia, Colombia. *Caribbean Journal of Science* 23:386–399.

Rivero JA. Serna MA. 1991. A new species of *Atelopus* (Amphibia, Bufonidae) from Antioquia, Colombia. *Brenesia* 36:15 20.

Rivero JA, Serna MA. 1991a. Tres nuevas especies de *Colostethus* (Anfibia, Dendrobatidae) de Colombia. *Trianea* 4:481–495.

Rivero JA, Serna MA, 1995. Nuevos *Colostethus* (Amphibia, Dendrobatidae) del Departamento de Antioquia, Colombia, con la descripción del renacuajo de *Colostethus fraterdanieli*. *Revista de Ecologia Latino Americana* 2:45-58

Roa Trujillo SH, Ruiz Carranza PM. 1991. Una nueva especie de *Eleutherodactylus* (Amphibia: Leptodactylidae) de la Cordillera Central de Colombia. *Caldasia* 16:343–348.

Roberto IJ, Cardozo D, Ávila RW. 2013. A new species of *Pseudopaludicola* (Anura, Leiuperidae) from Western Piauí State, Northeast Brazil. *Zootaxa* 3636:348–360.

Rojas Morales JA, Arias Monsalve HF, González Durán GA. 2014. Anfibios y reptiles de la región centro sur del departamento de Caldas, Colombia. *Biota Colombiana* 15:73 93.

Rojas Rivera MA, Gutiérrez-Cárdenas PDA, Cortés Bedoya S. 2013. *Pristimantis achatinus* (Boulenger 1898). *Catálogo de Anfibios y Reptiles de Colombia* 1:35-44.

Rojas-Runjaic FJ, Infante-Rivero EE, Salerno PE, Meza-Joya FL. 2018. A new species of *Hyloscirtus* (Anura, Hylidae) from the Colombian and Venezuelan slopes of Sierra de Perijá, and the phylogenetic position of *Hyloscirtus jahni* (Rivero, 1961). *Zootaxa* 4382:121-146.

Romero Garcia J, Clavijo Garzon S, Bernal MH. (2015). The advertisement call of three highland endemic species (Anura: Craugastoridae) from the Andean mountains of Colombia. *Zootaxa* 4007:298-300.

Ron SR, Mueses Cisneros JJ, Gutiérrez-Cárdenas PDA, Rojas Rivera MA., Lynch RL, Duarte Rocha CF, Galarza G. 2015 Systematics of the endangered toad genus *Andinophryne* (Anura: Bufonidae): phylogenetic position and synonymy under the genus *Rhaebo*. *Zootaxa* 3947:347–366.

Rueda Almonacid JV, Lynch JD. 1983. Una nueva especie de *Eleutherodactylus* (Amphibia: Leptodactylidae) para la Cordillera Oriental de Colombia. *Lozania, Acta Zoologica Colombiana* 42:1–6.

Rueda Almonacid JV, Lynch JD, Galvis-Peñuela PA. 2003. Una nueva especie de anfibio (Anura: Leptodactylidae) de los alrededores de La Sabana de Bogotá, Colombia. *Revista de la Academia Colombiana de Ciencias Exactas, Físicas y Naturales* 27:461–466.

Rueda Almonacid JV, Rada M, Sánchez-Pacheco SJ, Velásquez-Álvarez AA, Quevedo A. 2006. Two new and exceptional poison dart frogs of the genus *Dendobates* (Anura: Dendrobatidae) from the northeast flank of the Cordillera Central of Colombia. *Zootaxa* 1259:39-54.

Ruiz-Carranza PM, Ardila-Robayo MC. 1991. Una nueva especie de *Hyla* del grupo *bogotensis* (Amphibia: Anura: Hylidae) de la Cordillera Oriental de Colombia. *Caldasia* 16:337-342.

Ruiz-Carranza PM, Ardila-Robayo MC, Lynch JD, Restrepo-Toro JH. 1997. Una nueva especie de *Gastrotheca* (Amphibia: Anura: Hylidae) de la Cordillera Occidental de Colombia. *Revista de la Academia Colombiana de Ciencias Exactas, Físicas y Naturales* 21:373-378.

Ruiz-Carranza PM, Hernández-Camacho JI. 1976. *Gastrotheca andaquiensis*, nueva especie de la Cordillera Oriental de Colombia (Amphibia, Anura). *Caldasia* 11:149-158.

Ruiz Carranza PM, Hernández-Camacho JI. 1976a. *Osornophryne* género nuevo de anfibios bufónidos de Colombia y Ecuador. *Caldasia* 11:93-148.

Ruiz-Carranza PM, Hernández-Camacho JI. 1978. Una nueva especie colombiana de *Atelopus* (Amphibia: Bufonidae). *Caldasia* 12:181-197.

Ruiz-Carranza PM, Hernández-Camacho JI., Ardila-Robayo M.C. 1986. Una nueva especie colombiana del género *Centrolene* Jiménez de la Espada 1872 (Amphibia: Anura) y redefinición del género. *Caldasia* 15:431-444.

Ruiz-Carranza PM, Lynch JD. 1991. Ranas Centrolenidae de Colombia II. Nuevas especies de *Centrolene* de la Cordillera Oriental y Sierra Nevada de Santa Marta. *Lozania* 58:1 28.

Ruiz-Carranza PM, Lynch JD. 1991a. Ranas Centrolenidae de Colombia III. Nuevas especies de *Cochranella* del grupo *granulosa*. *Lozania* 59:1-18.

Ruiz-Carranza PM, Lynch JD. 1991b. Ranas Centrolenidae de Colombia IV. Nuevas especies de *Cochranella* del grupo *ocellata* de la Cordillera Oriental. *Lozania* 60:1-13.

Ruiz-Carranza PM, Lynch JD. 1995. Ranas Centrolenidae de Colombia V. Cuatro nuevas especies de *Centrolene* de la Cordillera Central. *Lozania* 62:1-23

Ruiz-Carranza PM, Lynch JD. 1995a. Ranas Centrolenidae de Colombia VI. Cuatro Nuevas especies de *Cochranella* de La Cordillera Occidental. *Lozania* 63:1-15.

Ruiz-Carranza PM, Lynch JD. 1995b. Ranas Centrolenidae de Colombia VII. Redescripcion de *Centrolene andinum* (Rivero 1968). *Lozania* 64:1-12.

Ruiz-Carranza PM, Lynch JD. 1995c. Ranas Centrolenidae de Colombia VIII. Cuatro nuevas especies de *Centrolene* de la Cordillera Central. *Lozania* 65:1-16.

Ruiz-Carranza PM, Lynch JD. 1996. Ranas Centrolenidae de Colombia IX. Dos nuevas especies del suroeste de Colombia. *Lozania* 68:1-11.

Ruiz-Carranza PM., Lynch JD. 1997. Ranas Centrolenidae de Colombia X. Los centrolénidos de un perfil del flanco oriental de la Cordillera Central en el Departamento de Caldas. *Revista de la Academia Colombiana de Ciencias Exactas, Físicas y Naturales* 21:542-553.

Ruiz-Carranza PM, Lynch JD. 1998. Ranas Centrolenidae de Colombia XI. Nuevas especies de ranas cristal del género *Hyalinobatrachium*. *Revista de la Academia Colombiana de Ciencias Exactas, Físicas y Naturales* 22:571-586.

Ruiz-Carranza PM, Lynch JD, Ardila Robayo MC. 1997. Seis nuevas especies de *Eleutherodactylus* Duméril & Bibron, 1841 (Amphibia: Leptodactylidae) del Norte de la Cordillera Occidental de Colombia. *Revista de la Academia Colombiana de Ciencias Exactas, Físicas y Naturales* 21:155–179.

Ruiz-Carranza PM, Osorno Muñoz M. 1994. Tres nuevas especies de *Atelopus* A.M.C. Dumeril and Bibron 1841 (Amphibia: Bufonidae) de la Cordillera Central de Colombia. *Revista de la Academia Colombiana de Ciencias Exactas, Físicas y Naturales* 19:165-179.

Ruiz-Carranza PM, Ramírez Pinilla M. 1992. Una nueva especie de *Minyobates* (Anura: Dendrobatidae) de Colombia. *Lozania* 61:1-15.

Ruiz-Carranza PM, Vélez Rodriguez CM, Ardila Robayo MC. 1995. Una nueva especie de *Atelopus* A. M. C. Dumeril & Bibron, 1841 (Amphibia: Bufonidae) de la Cordillera Occidental, Colombia. *Caldasia* 18:113–118.

Sánchez DA. 2010. Larval development and synapomorphies for species groups of *Hyloscirtus* Peters, 1882 (Anura: Hylidae: Cophomantini). *Copeia* 2010:351-363.

Sánchez DA. 2013 Larval morphology of Dart-Poison Frogs (Anura: Dendrobatoidea: Aromobatidae and Dendrobatidae). *Zootaxa* 3637:569–591.

Serna-Botero V, Ramírez-Castaño VA. 2017. Curaduría y potencial de investigación de la colección herpetológica del Museo de Historia Natural de la Universidad de Caldas, Manizales, Colombia. *Boletín Científico. Centro de Museos. Museo de Historia Natural* 21:138-153.

Sheil CA., Mendelson III JR. 2001. A new species of *Hemiphractus* (Anura: Hylidae: Hemiphractinae), and a redescription of *H. johnsoni*. *Herpetologica* 57:189-202.

Sheil CA, Mendelson III JR., Da Silva HR. 2001 Phylogenetic Relationships of the Species of Neotropical Horned Frogs, Genus *Hemiphractus* (Anura: Hylidae: Hemiphractinae), Based on Evidence from Morphology. *Herpetologica* 57:203-214

Silverstone PA. 1971. Status of certain frogs of the genus *Colostethus*, with descriptions of new species. *Contributions in Science. Natural History Museum of Los Angeles County* 215:1-8.

Silverstone PA. 1975. A revision of the poison arrow frogs of the genus *Dendrobates* Wagler. *Natural History Museum Los Angeles County Science* 21:1-53.

Silverstone PA. 1975a. Two new species of *Colostethus* (Amphibia: Anura: Dendrobatidae) from Colombia. *Contributions in Science. Natural History Museum of Los Angeles County* 268: 1-10.

Silverstone PA. 1976. A revision of the poison arrow frogs of the genus *Phyllobates* Bibron in Sagra (Family Dendrobatidae). *Natural History Museum of Los Angeles County Science Bulletin* 27:1-53.

Smith EN, Noonan BP. 2001. A new species of *Osteocephalus* (Anura: Hylidae) from Guyana. *Revista de Biología Tropical* 49:347-357.

Suárez Badillo HA, Ramírez Pinilla MP. 2004. Anuros del gradiente altitudinal de la Estación experimetal y demostrativa el Rasgón (Santander, Colombia). *Caldasia* 26:395-416.

Suárez-Mayorga AM. 1999. Lista preliminar de la fauna amphibia presente en el transecto La Montañita - Alto de Gabinete, Caqueta, Colombia. *Revista de la Academia Colombiana de Ciencias Exactas, Físicas y Naturale*s 23:395-405.

Trueb L. 1971. Phylogenetic relationships of certain Neotropical toads with the description of a new Genus (Anura: Bufonidae). *Natural History Museum of Los Angeles County Contributions in Science* 216:1-40.

Trueb L. 1974. Systematic relationships of neotropical horned frogs, genus *Hemiphractus* (Anura: Hylidae). *Occasional Papers of the Museum of Natural History, University of Kansas* 29:1-60.

Trueb L. 1984. Description of a new species of *Pipa* (Anura: Pipidae) from Panama. *Herpetologica* 40:225-234.

Trueb L, Duellman WE. 1971. A synopsis of neotropical hylid frogs, genus *Osteocephalus*. *Occasional Papers of the Museum of Natural History, University of Kansas* 1:1-47.

Andrade-C MG, Lynch JD. 2007. Los tipos nomenclaturales depositados en la colección zoológica del Instituto de Ciencias Naturales. Instituto de Ciencias Naturales, Facultad de Ciencias, Universidad Nacional de Colombia, Bogotá.

Valencia-Zuleta A, Jaramillo-Martínez AF, Yánez-Muñoz MH. 2016. Redescription, distribution and mating call of *Pristimantis colomai* (Lynch and Duellman, 1997) (Anura, Craugastoridae). *Zootaxa* 4193:590–594.

Vanegas Guerrero J, Gonzalez Duran GA, Escobar Lasso S. 2016. Distribution, diet, and vocalizations of the endangered Colombian Toad *Osornophryne percrassa* (Anura, Bufonidae). *Herpetological Conservation and Biology* 11:90 100.

Vargas F, Gutiérrez-Cárdenas PDA. (2005). Cambios morfológicos y mortalidad en embriones y renacuajos de *Agalychnis spurrelli* Boulenger (Anura: Hylidae). *Actualidades Biologicas* 27:189-202.

Velandia Perilla JH, Yusti Muñoz AP., Bolívar García W. 2011. Distribution extension of *Strabomantis ruizi* (Lynch, 1981) (Amphibia, Anura, Strabomantidae) from the department of Valle del Cauca, Colombia. *Check List* 7:635–636.

Velásquez A, Rada M, Sanchéz S, Acosta AR. 2007. A new species of glassfrog (Anura: Centrolenidae) from the western slope of Cordillera Oriental, Colombia. *South American Journal of Herpetology* 2:191-197.

Vélez Rodríguez. CM. 1999. Presencia de *Bufo sternosignatus* Günther 1859 (Amphibia: Anura: Bufonidae) en Colombia. *Revista de la Academia Colombiana de Ciencias Exactas, Físicas y Naturales* 23(suplemento): 411-416.

Vélez Rodríguez CM, Ruiz-Carranza PM. 1997. Una nueva especie de *Atelopus* (Amphibia: Anura: Bufonidae) de la Cordillera Central de Colombia. *Revista de la Academia Colombiana de Ciencias Exactas, Físicas y Naturales* 21:553-563.

Vélez Rodríguez CM, Ruiz-Carranza PM. 2002. A new species of *Bufo* (Anura: Bufonidae) from Colombia. *Herpetologica* 58:453 462.

Wild ER. 1994. Two new species of centrolenid frogs from the Amazonian slope of the Cordillera Oriental, Ecuador. *Journal of Herpetology* 28:299-310.
