# Supplementary figures and images for: What are the Andean Colombian anurans? Empirical regionalization proposals vs. observed patterns of compositional dissimilarity
Source: PeerJ. 2023 Jun 13;11:e15217. doi: 10.7717/peerj.15217 (PMC10274619; doi:10.7717/peerj.15217)

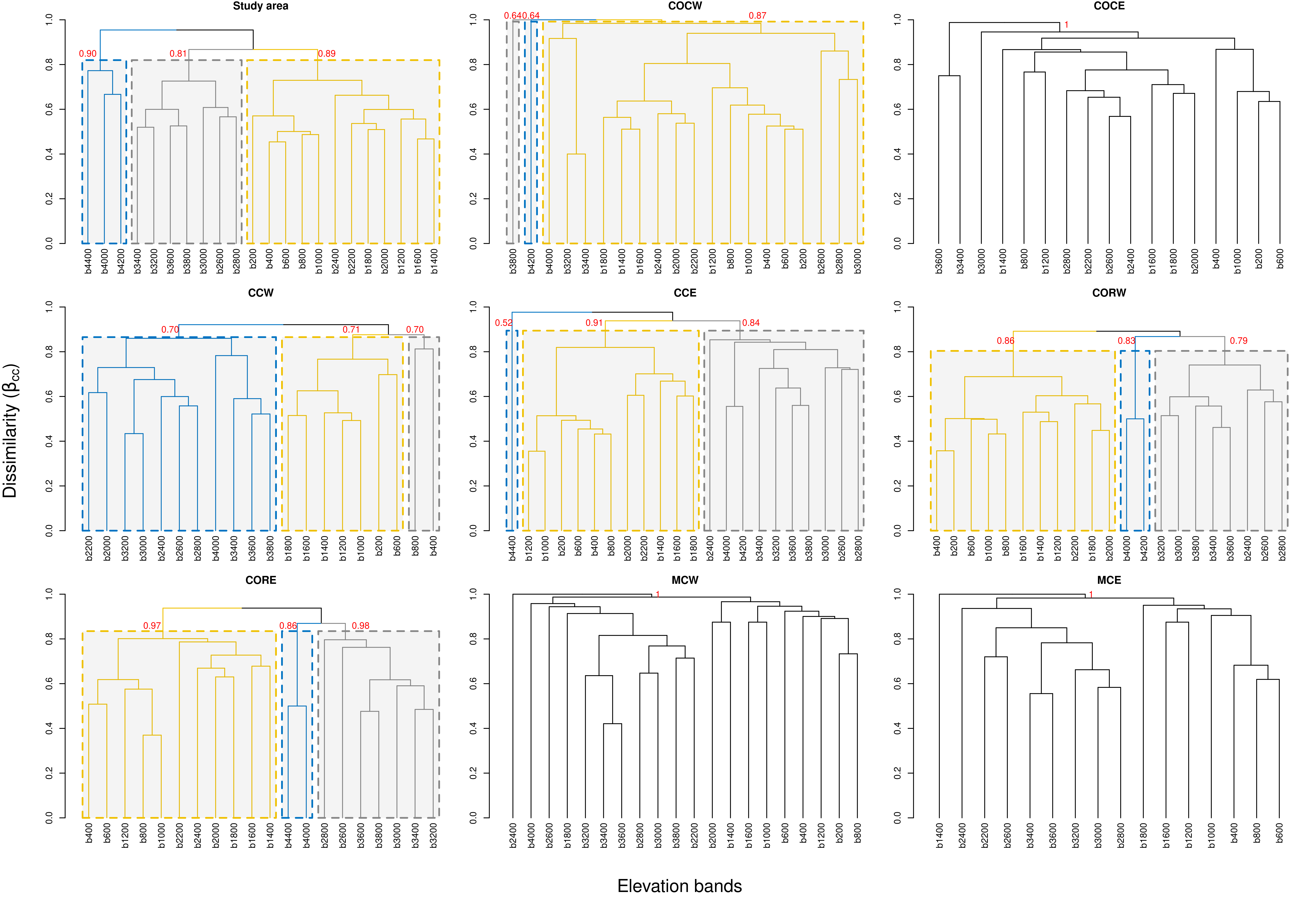

Supplement: Supplemental Information 2 — A. Complete Andes region. B. Western slope of Occidental Cordillera. C. Eastern slope of Occidental Cordillera. D. Western slope of Central Cordillera. E. Eastern slope of Central Cordillera. F. Western slope of Oriental Cordillera. G. Eastern slope of Oriental Cordillera. H. Western slope of the South block of the Colombian Andes. I. Eastern slope of the South block of the Colombian Andes. Values above each group correspond to their support using Jaccard’s Bootstrap (1,000 replicates). [file peerj-11-15217-s002.png]
