# Supplementary material for: What are the Andean Colombian anurans? Empirical regionalization proposals vs. observed patterns of compositional dissimilarity
Source: PeerJ. 2023 Jun 13;11:e15217. doi: 10.7717/peerj.15217 (PMC10274619; doi:10.7717/peerj.15217)
Supplement: Supplemental Information 4 [file peerj-11-15217-s004.doc]

**00. Information about the metadata files:**

**Manuscript title:** What are the Andean Colombian anurans? Empirical regionalization proposals vs. observed patterns of compositional dissimilarity

**DOI:**

**Contact:** [mario.herreralopera@gmail.com](mailto:mario.herreralopera@gmail.com) - [viviana.ramirez@ucaldas.edu.co](mailto:viviana.ramirez@ucaldas.edu.co) – [carlos.cultid@inecol.mx](mailto:carlos.cultid@inecol.mx)

**1. Table S1**: Sources per family. The file contains the literature sources from which the records used in the analysis were extracted (the part corresponding to the literature search). For complete information on the literature sources for each of the records please contact the authors.

**2. Figure S1**: Complete clusters of the study area and the different entities that compose it. The code to generate the figures can be found in the R code provided. The description of how the figures were created can be found in the data analysis section of the manuscript.

**3. Shapefile of the study area**. File in .shp format of the area described in the "study area" section. Each entity is differentiated within the attributes table. Its projection is EPSG4326 – WGS84. COCC: Cordillera Occidental, CC: Cordillera Central, COR: Cordillera Oriental, Macizo: Macizo Colombiano. Suffix “ori”: eastern slope, suffix “occi”: western slope.

**4. Records used for analysis**. File in .csv format with the unique localities of the anuran species used in the analysis. Columns: sp_cod_COR: species code in the analysis, sp_COR: updated species name according to Frost (2022); genus_COR: updated species genus according to Frost (2022); species_CO: updated species epithet according to Frost (2022); family_COR: updated family according to Frost (2022); sp_OR: species as it originally appears in the source (or if its source is literature, with taxonomy updated to Frost 2018); genus_OR: genus as it appears in the source (or if its source is literature, with taxonomy updated to Frost 2018); species_OR: epithet as it appears in the record (or if its source is literature, with taxonomy updated to Frost 2018); Family: Family as it appears in the source (or if its source is literature, with taxonomy updated to Frost 2018); Scientific: Scientific name of the species in its source (or if its source is literature, with taxonomy updated to Frost 2018); x: Longitude in hexadecimal coordinates (EPSG:4326 - WGS84); y: Latitude in hexadecimal coordinates (EPSG:4326 - WGS84); Source: Origin of the record (Literature or GBIF); Elevation: Elevation assigned (see methods section of the article); Region and subregion: Region to which the record belongs (COCC: Cordillera Occidental, CC: Cordillera Central, COR: Cordillera Oriental, Macizo: Macizo Colombiano. Suffix "ori": eastern slope, suffix "occi": western slope); group: Elevation band to which the record belongs.

**5. RCode:** Code in R language that can be used to replicate the analysis. This way:

**5.1 Dwn_records**: Download and clean records from GBIF: Download anuran records for Colombia from GBIF using the rgbif package (See materials and methods section of the article) eliminate records without species or genus and remove records of species introduced into the country.

**5.2 Synonyms**: Obtaining the current names of the species, their genera and families.

**5.3 Rec_curation**: Record curation: Clean up GBIF records and literature records and merge them into a single table.

**5.4 Bands:** Assigning to the records their elevation bands. Using QGIS, the clean records are taken and using the sampling point tool, the elevation, region and subregion are assigned. In this script the 200 m elevation bands are assigned to each record.

**5.5 Cluster**: Grouping data: Forming the clusters of elevation bands seen in Figure 2.

**5.6 Richness**: Maps of species richness and records richness. Extract the data required to construct Figure 4 (Finished in QGIS).

**5.7 Heatmap**: R code needed to create the heat-map mosaic that can be seen in Figure 2. The figure was finalized in Inkscape software.
